# The Role of Xanthine Oxidase in Hemodialysis-Induced Oxidative Injury: Relationship with Nutritional Status

**DOI:** 10.1155/2013/245253

**Published:** 2013-06-02

**Authors:** Dijana Miric, Bojana Kisic, Radojica Stolic, Bratislav Miric, Radoslav Mitic, Snezana Janicijevic-Hudomal

**Affiliations:** ^1^Institute of Biochemistry, Medical Faculty Pristina, 38220 Kosovska Mitrovica, Serbia; ^2^Internal Clinic, Medical Faculty Pristina, 38220 Kosovska Mitrovica, Serbia; ^3^Department of Informatics, State University of Novi Pazar, 36300 Novi Pazar, Serbia; ^4^Institute of Pharmacology, Medical Faculty Pristina, 38220 Kosovska Mitrovica, Serbia

## Abstract

The role of xanthine oxidase (XOD) in patients undergoing chronic hemodialysis treatment (HD) is poorly understood. Geriatric nutritional risk index (GNRI) ≤ 90 could be linked with malnutrition-inflammation complex syndrome. This study measured XOD, myeloperoxidase (MPO), superoxide dismutase (SOD), lipid hydroperoxides, total free thiol groups, and advanced oxidation protein products (AOPP) in 50 HD patients before commencing (pre-HD) and immediately after completion of HD session (post-HD) and in 22 healthy controls. Pre-HD serum hydroperoxides, AOPP, XOD, and SOD were higher and total thiol groups were lower in patients than in controls (*P* < 0.05, resp.). Compared to baseline values, serum MPO activity was increased irrespective of GNRI status. Serum XOD activity was increasing during HD treatment in the group with GNRI ≤ 90 (*P* = 0.030) whilst decreasing in the group with GNRI > 90 (*P* = 0.002). In a multiple regression analysis, post-HD serum XOD activity was independently associated with GNRI ≤ 90 (**β**  ± SE: 0.398 ± 0.151; *P* = 0.012) and HD vintage (**β**  ± SE: −0.349 ± 0.139; *P* = 0.016). These results indicate that an upregulated XOD may be implicated in HD-induced oxidative injury contributing to accelerated protein damage in patients with GNRI ≤ 90.

## 1. Introduction

Oxidative stress and malnutrition-inflammation complex syndrome often coexist in critically ill patients and have recently came into a focus as nontraditional risk factors of cardiovascular morbidity and overall mortality in patients with end-stage renal disease (ESRD) [1–5]. The reasons underlying chronically disturbed oxidant homeostasis in ESRD may include various factors, such as progressive deterioration of renal metabolic activities, inflammation, uremic toxins, and restrictive diets [1, 6, 7]. It has been previously shown that even a single hemodialysis (HD) treatment can provoke the formation of oxidants, which was largely attributed to activation of leukocytes by bio-incompatible dialysis membrane and release of myeloperoxidase (MPO) into the blood [2]. Extracellular MPO is well known to catalyze the peroxidation of the blood low-density lipoproteins (LDL) and albumin, leading to the formation of oxidized LDL and advanced oxidation protein products (AOPP), thereby contributing to augmentation of prooxidant and proinflammatory state in the vascular compartment [2, 4, 5].

Xanthine dehydrogenase (XDH) is a cytoplasmic enzyme implicated in hydroxylation of hypoxanthine to xanthine and its oxidation to uric acid and a relevant source of oxidants in vasculature [8–10]. XDH may undergo limited proteolysis or oxidation of critical cysteine residues to yield the xanthine oxidase (XOD) form. Unlike XDH that generates mostly superoxide anion radicals, XOD more efficiently catalyzes the formation of hydrogen peroxide, which is less reactive but long-lived oxidant. Previous studies have revealed systematically upregulated XOD in inflammation, diabetes, and cardiovascular diseases [11, 12]. Moreover, serum XOD activity was found to be markedly elevated in HD and peritoneal dialysis patients, independently of dialysis modality [13]. 

Patients undergoing HD treatment have high prevalence of malnutrition-inflammation syndrome, clinically presented as muscle and fat tissue wasting, loss of visceral proteins, and higher inflammatory and oxidative state [1, 4, 5]. There is currently no clear explanation about mechanisms underlying enhanced oxidative stress in HD patients with low nutritional status. However, recent experimental studies suggest that targeting XOD by allopurinol may protect against oxidative stress, inflammatory cytokine signaling, proteolytic activity, and tissue wasting [14]. Geriatric nutritional risk index (GNRI) is a useful tool for nutritional screening, based on simple anthropometric measures and serum albumin concentration. In a cohort of 490 chronic HD patients, the predialytic GNRI values below 90 have been recently linked with increased inflammatory CRP levels and mortality rates [3]. Given that prooxidant enzymes can play significant roles in oxidative stress, this study assessed serum MPO and XOD activities and oxidative stress markers in relation to nutritional status in ESRD patients on chronic HD treatment. 

## 2. Subjects and Methods

### 2.1. Study Participants

Fifty adults, clinically stabile nonsmoker ESRD patients (23 males, 27 females, mean age 57.4 ± 12.6 years) were enrolled in the study after written informed consent was provided. Patients were routinely dialyzed 12 hours per week in two local dialysis centers using commercially available dialysers in the bicarbonate hemodialysis and hemodiafiltration (Fresenius Medical Care, Bad Homburg, Germany). The causes of ESRD were diabetic nephropathy (28%), polycystic kidney disease (22%), chronic glomerulonephritis (16%), nephrosclerosis (12%) chronic pyelonephritis (8%), and nephropathy of unknown etiology (14%). Excluded were patients with known malignant, hepatic or autoimmune diseases, acute infections, or recent cardiovascular events. The control group was consisted of 22 age- and sex-matched healthy subjects (9 males, 13 females, mean age 58.4 ± 9.3 years). This study was conducted following the tenets of Declaration of Helsinki. Ethical clearance for the study was obtained from the Ethics Committee of Medical Faculty Pristina, Kosovska Mitrovica. 

### 2.2. Sample Collection

At the middle of dialysis week, 10 mL of blood was taken into tubes with or without EDTA, prior to anticoagulation and start of HD (pre-HD) and immediately after completion of HD treatment (post-HD). 

### 2.3. Biochemical Methods

#### 2.3.1. Measurement of Serum Xanthine Oxidase Activity

Serum XOD activity was determined by UV method, using xanthine as a substrate, as described in [15]. The formation of uric acid was continuously monitored at *λ* = 293 nm on an UV/VIS spectrophotometer equipped with a constant temperature cuvette compartment (SAFAS 2, Monaco). XOD activity was calculated after correction for preexisting uric acid using molar absorbance of *ε* = 1.26 × 10^4^ L × M^−1^ × cm^−1^. One unit of XOD activity was defined as 1 *μ*mol uric acid formed per minute at 37°C.

#### 2.3.2. Assessment of Extracellular Myeloperoxidase Activity

Serum MPO activity was measured in the system of 4-aminoantipyrine and phenol with hydrogen peroxide as a substrate, by monitoring the formation of pinkish colored quinoneimine at *λ* = 505 nm [16]. MPO activity was calculated using molar absorbance of *ε* = 1.3 × 10^4^ L × M^−1^ × cm^−1^. One unit of MPO activity was defined as amount of enzyme degrading 1 *μ*mol of hydrogen peroxide per minute, at 25°C.

#### 2.3.3. Assessment of Extracellular Superoxide Dismutase Activity

Superoxide dismutase (SOD) is an antioxidant enzyme that catalyzes the conversion of superoxide anion radicals to hydrogen peroxide and molecular oxygen. Serum SOD activity was determined by the rate of inhibition of adrenaline autooxidation to adrenochrome, continuously monitored at 25°C for 3 minutes at *λ* = 480 nm and calculated using the molar absorbance of *ε* = 4.02 × 10^3^ L × M^−1^ × cm^−1^ [17]. One unit of SOD activity was defined as the quantity of enzyme that inhibits autooxidation of 5 mmol adrenaline by 50%.

#### 2.3.4. Determination of Total Free Thiol Groups

Serum free thiol groups (SH) are implicated in nonenzymatic antioxidant defense and can serve as a marker of acute oxidative protein injury. Concentration of serum SH groups was determined with Ellman's reagent and calculated using molar absorbance of *ε* = 1.36 × 10^4^ L × mol^−1^ × cm^−1^ [18].

#### 2.3.5. Measurement of Blood Oxidative Stress Markers

We measured two oxidative stress markers: total hydroperoxides, indicating an ongoing lipid peroxidation (LPO) and advanced oxidation protein products (AOPP) representing a marker of protein oxidative injury. Concentration of total serum hydroperoxides was measured by the ferrous-oxidation xylenol-orange method (FOX), following reduction of preexisting peroxides with triphenylphosphine [19]. Plasma AOPP was measured by the method of Anderstam et al. [20]. The absorbance readings were taken at *λ* = 340 nm against reagent blank. Concentration of AOPP was calculated respective to chloramine-T standard, corrected for dilution factor.

#### 2.3.6. Other Hematological and Blood Biochemical Measurements and Calculations

Blood cell count and differential were measured in EDTA blood samples with the Onyx hematology analyzer (Beckman Coulter, Krefeld, Germany). Total proteins, albumin, urea, total cholesterol, HDL-cholesterol, triglyceride, uric acid, and C-reactive protein (CRP) were routinely measured on Hitachi 902 chemistry analyzer (Roche Diagnostics GmbH, Mannheim, Germany), according to manufacturer's instructions. LDL cholesterol was calculated using Friedewald's formula. Plasma atherogenic index was calculated as Log (triglycerides/HDL cholesterol) [21].

Post-HD values of biochemical variables were corrected for intradialytic decrease of plasma volume, according to Leypoldt et al. [22]. The following formula was applied: *X*_POST-corrected  _ = *X*_POST_ × [1 − (TP_POST_ − TP_PRE_)/TP_POST_], where *X* and TP denote the particular biochemical variable and total serum protein concentrations in post-HD (TP_POST_) and pre-HD samples (TP_PRE_), respectively. The quality of dialysis was assessed as urea-based Kt/V formula.

### 2.4. Anthropometric and Nutritional Indices

Body mass index (BMI) was calculated respective to predialytic body weight by the formula: BMI (kg/m^2^) = present body weight/height^2^. Geriatric nutritional risk index (GNRI) was applied to assess nutritional status, calculated as follows using predialytic values of variables: GNRI = (1.489 × serum albumin (g/L)) + (41.7 × present/ideal body weight) [3]. The GNRI ≤ 90 was considered to be low [3].

### 2.5. Statistical Methods

Statistical analyses were accomplished with statistical software package MedCalc, version 8.0, (MedCalc Software, Ostend, Belgium). Data distribution and homogeneity of variance were tested by Kolmogorov-Smirnov test. Data were presented as mean value ± SD, geometric mean and 95% confidence interval (CI) of the mean or frequencies. Differences between groups were tested by one-way ANOVA and Student's *t*-test for independent or paired samples, where appropriate; chi-square test was used to compare nonparametric data. Correlation analysis was accomplished by calculating the Pearson's correlation coefficient (*r*). Multiple regression analysis was used to examine the influence of multiple clinical and biochemical variables on XOD activity. The significance level was set at *P* < 0.05. 

## 3. Results

A total of 50 ESRD patients and 22 controls were included in the study. Basic clinical and biochemical data of patients and controls are summarized in Table 1. In comparison to controls, patients with ESRD had higher neutrophil leukocyte count, ferritin, and CRP, while lower blood hemoglobin levels (Table 1). 

Baseline levels of oxidative stress markers, prooxidant enzymes, and SOD activity in ESRD patients and control subjects are presented in Table 2. In comparison to control values, serum total hydroperoxides and AOPP were significantly higher, and total SH groups were lower in patients. Also, extracellular SOD and XOD activities were at the baseline higher in patients than in controls, while MPO activity was comparable to control values (Table 2). 

**Figure 1:**
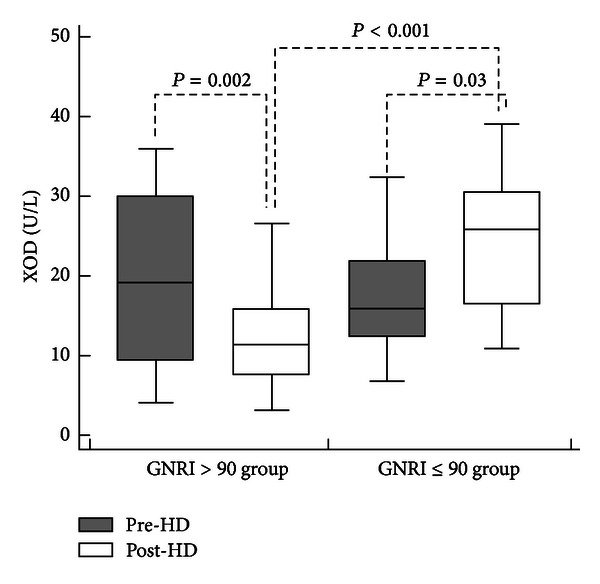
The impact of a single hemodialysis session on serum XOD activity. Serum xanthine oxidase activity (XOD) was determined prior to (pre-HD) and immediately after completion of hemodialysis session (post-HD) in patients with normal geriatric nutritional risk index (GNRI > 90) and with GNRI ≤ 90. In contrast to GNRI > 90 group, where a single HD session induced the fall of serum XOD activity from 19.3 ± 10.6 U/L to 12.1 ± 6.0 U/L (*P* = 0.002, paired samples *t*-test), in the group with GNRI ≤ 90, there was an elevation of serum XOD activity from 18.4 ± 7.8 U/L to 24.8 ± 7.3 U/L (*P* = 0.030, paired samples *t*-test), contributing to significant post-HD differences between GNRI groups (*P* < 0.001, independent samples *t*-test).

According to adopted criterion [3], ESRD patients were further divided into group with GNRI ≤ 90 (*n* = 15) and group with GNRI > 90 (*n* = 35). Seven patients in each group had diabetic nephropathy (chi-square = 2.499; *P* = 0.114). There was also no significant difference between GNRI > 90 and GNRI ≤ 90 groups regarding baseline concentrations of serum hydroperoxides and AOPP, as well as XOD and MPO activities (Table 3, Figure 1). However, baseline serum total SH groups were significantly lower, whilst SOD activity was higher in GNRI ≤ 90 than in GNRI > 90 group (Table 3).

To evaluate the impact of a single HD session on blood oxidants and antioxidants, all post-HD values were corrected for intradialytic decrease of blood plasma volume [22]. In comparison to baseline values, post-HD serum hydroperoxides and total SH groups were lower, while AOPP levels were unchanged. Relative to pre-HD values, serum SOD and MPO activities were higher in post-HD samples. The post-HD serum MPO activity was correlated with AOPP (*r* = 0.355; *P* = 0.011).

In comparison to group with GNRI > 90, post-HD serum hydroperoxides were higher and total SH groups were lower than in the group with  GNRI ≤ 90 (Table 3). Post-HD XOD activity also differed between groups (Figure 1), in such way that in GNRI > 90 group baseline serum XOD activity declined during HD from 19.3 ± 10.6 U/L to 12.1 ± 6.0 U/L (*P* = 0.002, paired samples *t*-test) while increased after HD treatment in GNRI ≤ 90 group from 18.4 ± 7.8 U/L to 24.8 ± 7.3 U/L (*P* = 0.030, paired samples *t*-test). Post-HD serum XOD activity was correlated with hydroperoxides (*r* = 0.540; *P* < 0.001), AOPP (*r* = 0.324; *P* = 0.022), and total SH groups (*r* = −0.578; *P* < 0.001).

We also modeled a multiple regression analysis to examine the influence of age, gender (male versus female), HD vintage, the quality of HD, presence of hypertension or diabetes (yes versus no, resp.), inflammatory CRP levels, plasma atherogenic index, and GNRI status (GNRI ≤ 90 versus GNRI > 90) on post-HD serum XOD activity. Univariate analysis (Table 4) showed that post-HD serum XOD activity was significantly correlated with HD vintage (*P* = 0.016), serum CRP (*P* = 0.028), and GNRI status (*P* = 0.001). In a multivariate regression with stepwise elimination mode, HD vintage (*P* = 0.016) and GNRI status (*P* = 0.012) were retained as independent predictors of post-HD serum XOD activity (Table 4).

## 4. Discussion

In the present study, we evaluated the relationship between serum XOD activity, oxidative stress markers, and nutritional status in HD patients. The major finding was that elevation of XOD activity during a single HD session was positively correlated with serum hydroperoxides and AOPP and independently associated with GNRI ≤ 90, as an indicator of poor nutritional status. These results suggest that an upregulated XOD may exacerbate oxidative injury during HD treatment contributing to pathogenesis of malnutrition-inflammation complex syndrome. 

Chronic uremia is known to induce a large-scale oxidative modifications of blood lipids and proteins, leading to increased hydroperoxides and AOPP and loss of free SH groups [2, 6, 7], as was also observed in the current study. The majority of serum free SH groups are provided by albumin, whose single free SH group at cysteine 34 is exposed at the surface of the molecule. Acting as a sacrificial antioxidant albumin can prevent oxidative damage of practically all blood constituents, which is particularly important in cases when other antioxidants, such as vitamin C, are present at chronically low levels or at highly oxidized state. However, the oxidation of free SH and other critical groups may facilitate albumin fragmentation and subsequent breakdown [23, 24] and further deteriorate the blood protein, nutritional and antioxidant status. Moreover, some blood antioxidant and anti-inflammatory proteins, such as *α*_1_-antitrypsin and HDL apolipoprotein A, were found to be extensively oxidized in ESRD patients with malnutrition-inflammation complex syndrome [4, 5]. 

Despite of the fact that the maintenance HD is currently the major treatment modality in ESRD, there are many controversies of whether and how it influences the burden of oxidatively modified molecules. Previous studies have demonstrated both the fall of serum hydroperoxides, as early as the first hour of HD session, and a significant increase of LPO adducts and AOPP in post-HD samples [6, 7]. In the current study serum hydroperoxides were markedly reduced during HD, while AOPP remained virtually unchanged (Table 3). Such findings could reflect different diffusion rates of hydroperoxides and macromolecular AOPP into dialytic fluid or faster decomposition of hydroperoxides to other LPO adducts that could not be detected with xylenol orange test. We observed that in comparison to the group with normal GNRI, those with low GNRI values had higher serum hydroperoxides and lower total SH groups after completion of HD treatment, which may indicate a higher degree of oxidative damage imposed during HD associated with worse blood nutritional status. 

Aside from chronic uremia-induced oxidative stress, ESRD patients usually endure an intermittent oxidative injury during each HD treatment. It is generally believed that the major reason is activation of leukocytes upon contact with bio-incompatible dialysis membrane or impurity in dialysis fluid and release of MPO into the blood [1, 2]. Extracellular MPO is a powerful catalyst of the LPO process and induce chlorination and nitrosylation of various blood compounds, giving rise to dysfunctional molecules, toxic mediators, atherogenic lipids and protein oxidation products, such as AOPP [2]. Accordingly, serum MPO activity was by 300% increased after completion of HD and positively correlated with AOPP (*r* = 0.355; *P* = 0.011), which is consistent with evidence that dityrosine-containing oxidized albumin is the main constituent of AOPP [2, 25]. However, in agreement to some previous studies [4, 26], baseline serum MPO activity was in ESRD patients similar to that in healthy controls and comparable between GNRI groups (Table 2), and the post-HD MPO activities do not differ between patients' groups (Table 3).

On the other side, the baseline serum XOD activity was in ESRD patients far over the values in healthy subjects (Table 2) and in agreement with Choi et al. [13]. However, this finding may be rather expected having in mind that a variety of stimuli usually present in ESRD, like endotoxemia or hypoxia, can enhance the transcriptional activity of the XOD gene [9]. Beside controlling purine catabolic pathway, the XOD can induce the expression of cyclooxygenase-2 [27], translocation of nuclear factor-*κ*B, synthesis of TNF-*α*, activation of neutrophils, and phagocytic killing [8, 12], being therefore a potent modulator of innate immune response. In turn, inflammatory cytokines may be responsible for upregulated synthesis of both CRP and XOD [28], and a positive correlation between these parameters in the current study (Table 4) further supports the idea that XOD has a putative role in inflammatory signal transduction [8].

XDH/XOD is constitutively expressed in endothelial and many other cells. Still, the majority of vascular enzyme are most probably of hepatic origin [9] and are reversibly attached to endothelial cell surface via glycosaminoglycan-rich receptors. The release of extracellular XOD into circulation occurs upon competitive binding of heparin, proportionally to the content of vascular wall enzyme [10]. This was used for determination of endothelial XOD *in vivo*, particularly in pathologies associated with endothelial dysfunction. For example, a bolus injection of heparin (5000 IU) has been shown to induce an increase of serum XOD activity by 200% within a few minutes in patients with chronic heart failure but not in healthy controls [23]. In the current study all ESRD patients were routinely anticoagulated with unfractionated heparin solution, in accordance to European best practice guidelines. Taking into account the results of previous studies [10, 23], we may speculate that the post-HD elevation of serum XOD activity in the group with low GNRI status could reflect the basically higher content of endothelium-bound XOD. 

Endothelial cells also contain some SOD enzyme bound to the cell surface, which probably serves to counterbalance the local formation of oxidants and is also released into the blood after bolus injection of heparin [23]. In the current study, there were differences regarding absolute values and the direction in which serum XOD activities have changed during HD, although the rise of SOD activity was almost equal in both GNRI groups. These results suggest that a poor nutritional status in ESRD could be associated with an imbalanced presence of prooxidant and antioxidant enzymes in the vascular compartment. 

Patients undergoing HD treatment often experience some degree of intradialytic hypoxia associated with volume overload that may extend between two HD sessions [22]. During hypoxic periods, there is a decrease of intracellular pH leading to facilitated conversion of XDH to XOD form [9], while breakdown products, inosine and hypoxanthine, may accumulate due to poor regeneration of ATP. Hypoxanthine is normally present in plasma at only 1-2 *μ*M but is several fold increased in HD patients [13] and can serve as a substrate for XOD-catalyzed formation of oxidants in the blood. *Ex vivo* incubation of human plasma with 5 mU/mL XOD and 500 *μ*M hypoxanthine has been shown to oxidize up to 50% of total SH groups, mostly within the first 20 minutes [24]. According to our results, one of the reasons of enhanced oxidative damage in patients with low GNRI status could be increased presence of vascular XOD and/or the conversion of enzyme to oxidase form due to more severe hypoxia. 

XOD is present in the blood almost entirely at oxidase form but has a relatively short half-life of 2-3 hours [9]. However, in circulation, this enzyme retains the ability to prime naïve phagocytic cells [4] and produce oxidants [10, 12], especially at loci exposed to mechanical forces, such as oscillatory shear stress [29], causing activation of inflammatory cascade, deprivation of nitric oxide, and vascular dysfunction. In addition, the circulating XOD can be distributed to remote tissues, and after internalization into vascular and other cells, it may further exert pathological effects [30]. Moreover, targeting XOD with allopurinol has been shown to attenuate oxidative stress, cytokine signaling, and muscle wasting in a rat model of cancer cachexia [14] and inhibit MAPKinase signaling and ubiquitin-proteosome pathway thereby preventing atrophy and proteolysis in disused muscles [31]. The second major source of serum XOD in humans is gastrointestinal tract. The gut hypoperfusion or ischemic/reperfusion injury, prolonged parenteral nutrition, or poor nutritional status may upregulate XOD, which increases the permeability of intestinal barrier and mediates transmigration of bacteria (and endotoxin) into the blood, contributing to sustained microinflammatory state in ESRD [32]. Inflammation is highly prevalent in HD patients, and although its sources are not fully elucidated, it may lead to accelerated atherosclerosis. Moreover, murine macrophages overexpressing XOD have been shown to differentiate to foam cells [12], implicated in atherosclerotic plaque development.

The results from experimental studies imply that hyper- and dyslipidemia and cholesterol-rich diets can create a positive feedback loop and profoundly affect XOD activity, thereby linking lipid abnormalities with high expression of XOD [11, 12]. The present study found no significant relationship between serum XOD activity and atherogenic index of plasma, as a surrogate marker of atherogenic dyslipidemia [21]. Instead, the post-HD serum XOD activity was independently associated with low GNRI status (*β* ± SE: 0.398 ± 0.151; *P* = 0.012). Given that concentration of serum albumin is included in calculation of GNRI score, these results suggest that XOD can be implicated in protein oxidative injury during HD treatment. Due to chronic inflammation, uremia, loss of amino acids, restrictive diets or anorexia, and albumin synthesis may be insufficient to compensate for enhanced catabolic rate, and damage imposed by XOD-derived oxidants would probably enhance its breakdown, leading overtime to evident hypoalbuminemia.

## 5. Conclusions 

Taken together, these results indicate that an upregulated XOD can be implicated in protein oxidative damage and inflammatory cascade in ESRD patients. Repetitive liberation of XOD into the blood during each HD treatment could contribute to augmented oxidative damage and pathogenesis of inflammation-malnutrition complex syndrome. In ageing world population, there is increasing number of patients requiring maintenance HD treatment, and targeting factors associated with oxidative injury in ESRD may have medical, social, and economic aspects. Therefore, further studies are needed to evaluate the long-term relationships between XOD, oxidative stress, and nutritional status in patients on chronic HD treatment.

## Figures and Tables

**Table 1:** Basic clinical and biochemical data of ESRD patients and controls.

	Control group (*n* = 22)	ESRD patients (*n* = 50)
Age (years)	58.9 ± 13.3	61.7 ± 10.7
Gender (male/female, *n*)	9/13	14/14
Hypertension (yes/no, *n*)	8/14	8/14
Hemodialysis vintage (months)	NA	46.3 ± 36.6
*Kt*/*V* urea	NA	1.10 ± 0.37
Neutrophil leukocytes (10^9^/L)	4.10 ± 1.92	4.89 ± 2.07
Lymphocytes (10^9^/L)	2.21 ± 0.55	2.39 ± 0.92
Log CRP (mg/L)	0.114 ± 0.335	0.534 ± 0.633*
Albumin (g/L)	45.6 ± 5.1	40.6 ± 7.3*
Hemoglobin (g/L)	133.7 ± 20.2	118.6 ± 24.1*
Ferritin (*μ*g/L)	85 ± 62	511 ± 647*
Body mass index (kg/m^2^)	22.9 ± 5.3	21.4 ± 4.4
GNRI	114.3 ± 16.0	105.9 ± 13.4
Total cholesterol (mmol/L)	4.65 ± 1.14	4.90 ± 0.96
HDL cholesterol (mmol/L)	1.76 ± 0.54	1.54 ± 0.51
LDL cholesterol (mmol/L)	2.75 ± 0.65	3.09 ± 0.62
Triglycerides (mmol/L)	1.92 ± 0.91	2.13 ± 0.77
Plasma atherogenic index	0.152 ± 0.175	0.196 ± 0.172

*P < 0.05 versus controls; NA: not applicable.

**Table 2:** Blood oxidative stress markers and antioxidants in ESRD patients and controls.

	Control group (*n* = 22)	ESRD patients (*n* = 50)
Hydroperoxides (*μ*mol/L)	6.1 ± 1.5	12.5 ± 4.1*
AOPP (*μ*mol/L)	40.1 ± 11.1	91.3 ± 24.9*
Total SH groups (*μ*mol/L)	462 ± 73	371 ± 59*
SOD (kU/L)	43.6 ± 12.5	61.3 ± 17.1*
MPO (U/L)	16.9 ± 6.5	18.1 ± 5.4
XOD (U/L)	3.7 ± 1.0	18.5 ± 10.6*

*P < 0.05 versus controls.

**Table 3:** Comparisons of blood oxidative stress markers, MPO and SOD, activities before and after completion of hemodialysis session.

	GNRI > 90 group (*n* = 35)		GNRI ≤ 90 group (*n* = 15)	
	Pre-HD	Post-HD	Pre-HD	Post-HD
Hydroperoxides (*μ*mol/L)	12.9 ± 3.7	5.8 ± 1.7*	11.9 ± 3.0	7.9 ±2.8^∗,¶^
AOPP (*μ*mol/L)	94.6 ± 30.6	95.3 ± 28.7	82.2 ± 29.4	90.4 ± 27.5
Total thiol groups (*μ*mol/L)	371 ± 59	292 ± 60*	296 ±67^¶^	193 ±62^∗,¶^
MPO (U/L)	18.2 ± 6.9	71.3 ± 22.1*	19.6 ± 9.1	68.5 ± 25.9*
SOD (kU/L)	53.4 ± 20.4	101.3 ± 12.7*	75.6 ± 25.2*	107.1 ± 17.2*

Data are mean value ± SD. Differences between GNRI groups or between pre-HD and post-HD values were tested by independent samples *t*-test or paired samples *t*-test, respectively.

*P < 0.05 post-HD versus correspondent pre-HD value: ^¶^ *P* < 0.05 GNRI ≤ 90 versus GNRI > 90 group, at the same sampling time.

**Table 4:** Univariate and multivariate regression modeling predicting postdialytic serum XOD activity in ESRD patients.

Independent predictors	Univariate correlations			Multivariate model		
	β	SEM	*P* value	β	SEM	P value
Age (years)	0.008	0.144	0.953	−0.039	0.138	0.778
Gender (male versus female)	0.220	0.141	0.125	0.151	0.137	0.277
Hypertension (yes versus no)	−0.159	0.142	0.269	−0.044	0.135	0.742
Hemodialysis vintage (months)	−0.337	0.136	0.016	−0.349	0.139	0.016
Diabetes (yes versus no)	0.192	0.142	0.183	0.082	0.129	0.526
*Kt*/*V* urea	0.052	0.144	0.722	0.085	0.155	0.586
Log CRP (mg/L)	0.310	0.137	0.028	0.247	0.143	0.091
Plasma atherogenic index	0.116	0.143	0.424	0.175	0.128	0.225
GNRI ≤ 90 versus GNRI > 90	0.453	0.129	0.001	0.398	0.151	0.012

Multivariate *R* ^2^-adjusted = 0.278; *P* = 0.006.

## References

[1] Kalantar-Zadeh K, Balakrishnan VS (2006). The kidney disease wasting: inflammation, oxidative stress, and diet-gene interaction. *Hemodialysis International*.

[2] Capeillère-Blandin C, Gausson V, Nguyen AT, Descamps-Latscha B, Drüeke T, Witko-Sarsat V (2006). Respective role of uraemic toxins and myeloperoxidase in the uraemic state. *Nephrology Dialysis Transplantation*.

[3] Kobayashi I, Ishimura E, Kato Y (2010). Geriatric Nutritional Risk Index, a simplified nutritional screening index, is a significant predictor of mortality in chronic dialysis patients. *Nephrology Dialysis Transplantation*.

[4] Honda H, Ueda M, Kojima S (2009). Assessment of myeloperoxidase and oxidative a1-antitrypsin in patients on hemodialysis. *Clinical Journal of the American Society of Nephrology*.

[5] Honda H, Ueda M, Kojima S (2010). Oxidized high-density lipoprotein is associated with protein-energy wasting in maintenance hemodialysis patients. *Clinical Journal of the American Society of Nephrology*.

[6] Kuppusamy UR, Indran M, Ahmad T, Wong SW, Tan SY, Mahmood AA (2005). Comparison of oxidative damage in Malaysian end-stage renal disease patients with or without non-insulin-dependent diabetes mellitus. *Clinica Chimica Acta*.

[7] Obara M, Hirayama A, Gotoh M (2007). Elimination of lipid peroxide during hemodialysis. *Nephron Clinical Practice*.

[8] Gibbings S, Elkins ND, Fitzgerald H (2011). Xanthine oxidoreductase promotes the inflammatory state of mononuclear phagocytes through effects on chemokine expression, peroxisome proliferator-activated receptor-*γ* sumoylation, and HIF-1*α*. *Journal of Biological Chemistry*.

[9] Pacher P, Nivorozhkin A, Szabó C (2006). Therapeutic effects of xanthine oxidase inhibitors: renaissance half a century after the discovery of allopurinol. *Pharmacological Reviews*.

[10] Houston M, Estevez A, Chumley P (1999). Binding of xanthine oxidase to vascular endothelium: kinetic characterization and oxidative impairment of nitric oxide-dependent signaling. *Journal of Biological Chemistry*.

[11] Gwinner W, Scheuer H, Haller H, Brandes RP, Groene H (2006). Pivotal role of xanthine oxidase in the initiation of tubulointerstitial renal injury in rats with hyperlipidemia. *Kidney International*.

[12] Kushiyama A, Okubo H, Sakoda H (2012). Xanthine oxidoreductase is involved in macrophage foam cell formation and atherosclerosis development. *Arteriosclerosis, Thrombosis, and Vascular Biology*.

[13] Choi JY, Yoon YJ, Choi HJ (2011). Dialysis modality-dependent changes in serum metabolites: accumulation of inosine and hypoxanthine in patients undergoing hemodialysis. *Nephrology Dialysis Transplantation*.

[14] Springer J, Tschimer A, Hartman K (2012). Inhibition of xanthine oxidase reduces wasting and improves outcome in a rat model of cancer cachexia. *International Journal of Cancer*.

[15] Roussos GG (1967). Xanthine oxidase from bovine small intestine. *Methods in Enzymology*.

[16] Metcalf JA, Gallin JI, Nauseef WM, Root RK (1986). Myeloperoxidase functional assays. *Laboratory Manual of Neutrophil Function*.

[17] Misra HP, Fridovich I (1972). The role of superoxide anion in the autoxidation of epinephrine and a simple assay for superoxide dismutase. *Journal of Biological Chemistry*.

[18] Beutler E, Duron O, Kelly BM (1963). Improved method for the determination of blood glutathione. *The Journal of Laboratory and Clinical Medicine*.

[19] Nourooz-Zadeh J, Tajaddini-Sarmadi J, Ling KLE, Wolff SP (1996). Low-density lipoprotein is the major carrier of lipid hydroperoxides in plasma: relevance to determination of total plasma lipid hydroperoxide concentrations. *Biochemical Journal*.

[20] Anderstam B, Ann-Christin B, Valli A, Stenvinkel P, Lindholm B, Suliman ME (2008). Modification of the oxidative stress biomarker AOPP assay: application in uremic samples. *Clinica Chimica Acta*.

[21] Dobiášová M, Frohlich J (2001). The plasma parameter log (TG/HDL-C) as an atherogenic index: correlation with lipoprotein particle size and esterification rate inapob-lipoprotein-depleted plasma (FER_HDL_). *Clinical Biochemistry*.

[22] Leypoldt JK, Cheung AK, Delmez JA (2002). Relationship between volume status and blood pressure during chronic hemodialysis. *Kidney International*.

[23] Landmesser U, Spiekermann S, Dikalov S (2002). Vascular oxidative stress and endothelial dysfunction in patients with chronic heart failure: role of xanthine-oxidase and extracellular superoxide dismutase. *Circulation*.

[24] Radi R, Bush KM, Cosgrove TP, Freeman BA (1991). Reaction of xanthine oxidase-derived oxidants with lipid and protein of human plasma. *Archives of Biochemistry and Biophysics*.

[25] Witko-Sarsat V, Friedlander M, Khoa NT (1998). Advanced oxidation protein products as novel mediators of inflammation and monocyte activation in chronic renal failure. *Journal of Immunology*.

[26] Demirci Ş, Şekeroğlu MR, Noyan T (2011). The importance of oxidative stress in patients with chronic renal failure whose hypertension is treated with peritoneal dialysis. *Cell Biochemistry and Function*.

[27] Ohtsubo T, Rovira II, Starost MF, Liu C, Finkel T (2004). Xanthine oxidoreductase is an endogenous regulator of cyclooxygenase-2. *Circulation Research*.

[28] Moshage HJ, Janssen JAM, Franssen JH (1987). Study of the molecular mechanism of decreased liver synthesis of albumin in inflammation. *Journal of Clinical Investigation*.

[29] McNally JS, Davis ME, Giddens DP (2003). Role of xanthine oxidoreductase and NAD(P)H oxidase in endothelial superoxide production in response to oscillatory shear stress. *American Journal of Physiology*.

[30] Biffl WL, Moore EE (1996). Splanchnic ischaemia/reperfusion and multiple organ failure. *British Journal of Anaesthesia*.

[31] Derbre F, Ferrando B, Gomez-Cabrera MC (2012). Inhibition of xanthine oxidase by allopurinol prevents skeletal muscle atrophy: role of p38 MAPKinase and E3 ubiquitin ligases. *PloS ONE*.

[32] Wang F, Jiang H, Shi K, Ren Y, Zhang P, Cheng S (2012). Gut bacterial translocation is associated with microinflammation in end-stage renal disease patients. *Nephrology*.

